# Isolation and Biochemical Characterization of Recombinant Transketolase from Mycobacterium tuberculosis

**DOI:** 10.32607/actanaturae.11713

**Published:** 2022

**Authors:** T. A. Shcherbakova, S. M. Baldin, M. S. Shumkov, I. V. Gushchina, D. K. Nilov, V. K. Švedas

**Affiliations:** Lomonosov Moscow State University, Belozersky Institute of Physicochemical Biology, Moscow, 119991 Russia; Federal Research Centre «Fundamentals of Biotechnology», Russian Academy of Sciences, Moscow, 119071 Russia; Lomonosov Moscow State University, Faculty of Bioengineering and Bioinformatics, Moscow, 119991 Russia

**Keywords:** transketolase, thiamine diphosphate, xylulose 5-phosphate, ribose 5-phosphate, mycobacteria

## Abstract

Transketolase, an enzyme of the pentose phosphate pathway, plays an important role in the functioning of mycobacteria. Using plasmid pET-19b carrying the *Rv1449c *gene of transketolase from* Mycobacterium tuberculosis *and an additional histidine tag, we isolated and purified recombinant transketolase and determined the conditions for obtaining the apoform of the protein. The Michaelis constants were evaluated for the thiamine diphosphate cofactor in the presence of magnesium and calcium ions. We found that the affinity of mycobacterial transketolase for thiamine diphosphate is by three orders of magnitude lower than that of the human enzyme. Analysis of the structural organization of the active centers of homologous enzymes showed that this difference is due to a replacement of lysine residues by less polar amino acid residues.

## INTRODUCTION

Tuberculosis is a common infectious disease caused by *Mycobacterium tuberculosis*. Despite the centuries- long fight against tuberculosis, there are still no drugs that provide quick and safe treatment of this infectious disease. Therefore, the search for new molecular targets important for the vital function of mycobacteria and the development of selective inhibitors remain a priority. A genomic analysis of the H37Rv strain [1] has made it possible to identify the key biosynthetic processes involved; among them, the pentose phosphate pathway of carbohydrate metabolism is worth mentioning.

Transketolase (TK; [EC 2.2.1.1]) is a crucial enzyme of the pentose phosphate pathway that is involved in ketose (the donor substrate) cleavage and the subsequent transfer of a two-carbon fragment to aldose (the acceptor substrate). The enzyme is found in almost all animal and plant tissues, as well as in many microorganisms [2, 3, 4]. There are reasons to believe that *M. tuberculosis *TK (mbTK) participates in the synthesis of the carbohydrates that form the bacterial cell wall [5]. However, the biological properties of mbTK are still poorly understood, which makes difficult a search for effective enzyme inhibitors. Preliminary data on mbTK substrate specificity have been published, and a crystal structure has been determined (PDB ID 3rim) [6]. The aim of this study was to obtain a purified recombinant mbTK, characterize it biochemically, and study enzyme binding with the cofactor thiamine diphosphate (TDP) and the substrates xylulose 5-phosphate (X5P) and ribose 5-phosphate (R5P).

## EXPERIMENTAL

Recombinant mbTK was obtained using the* Escherichia coli *strain BL21(DE3). Cells were transformed using a pET-19b plasmid carrying the* Rv1449c *gene with a histidine tag and the ampicillin resistance gene. The transformed strain was grown in a LB medium for 12 h, transferred to a shake flask with a medium containing ampicillin (100 µg/mL) and incubated for 6–7 h at 180 rpm and 37°C. Expression of mbTK was initiated by lowering the temperature to 15°C, adding either MgCl_2_ or CaCl_2_ (2 mM), TDP (2 mM), isopropyl-β-*D*-1-thiogalactopyranoside (0.2 mM), and glycerol (2% v/v); the expression was conducted for 24 h. The cells were pelleted by centrifugation for 15 min at 4,000 g and 4°C, then resuspended in a phosphate buffer (50 mM NaH_2_PO_4_, pH 8.0; 0.3 M NaCl). After this, lysozyme (1 mg/mL) was added and the solution was incubated for 30 min. The cells were sonicated at 0°C. The resulting lysate was centrifuged for 30 min at 12,000 g and 4°C. The mbTK protein containing the decahistidine fragment was purified using Protino Ni-TED 1000 kit columns (Macherey-Nagel), according to the manufacturer’s instructions. The purity of the resulting mbTK sample was analyzed by polyacrylamide gel electrophoresis [7].

The activity of mbTK was measured by the coupled reaction of NAD^+^ reduction, catalyzed by glyceraldehyde 3-phosphate dehydrogenase from rabbit muscle [8]. The composition of the reaction system for measuring activity at pH 7.6 and 25°C was as follows: glyceraldehyde 3-phosphate dehydrogenase (3 U), glycylglycine (50 mM), dithiothreitol (3.2 mM), sodium arsenate (10 mM), magnesium or calcium chloride (2.5 mM), TDP (200 µM), X5P (500 µM), R5P (2,800 µM), and NAD^+^ (370 µM). The reaction was initiated by adding the mbTK solution. The reaction rate was monitored as an increase in the optical density of the solution for 3–5 min at 340 nm. A Shimadzu UV-1800 spectrophotometer was used to measure the reaction rate.

In order to generate the apo form of mbTK, the cofactors were removed according to the technique described in [9]. A saturated solution of ammonium sulfate (pH 3.5) was added to the mbTK holoenzyme solution (0.2 mg/mL) in a 10 mM glycylglycine buffer (pH 7.4) at a 2 : 3 ratio. The mixture was incubated on ice for 5 min and then centrifuged for 15 min at 12,000 g and 4°C. The precipitated protein was dissolved in a 50 mM glycylglycine buffer (pH 7.4). In order to determine the TDP binding constant, the mbTK apoenzyme was incubated in a 50 mM glycylglycine buffer (pH 7.6) in the presence of a 2.5 mM divalent cation (either Mg^2+^ or Ca^2+^) and TDP at different concentrations (0–200 μM) for 45–0 min at 25°C. Bovine serum albumin (1 mg/mL) was added to the sample to stabilize the protein. Other components necessary for measuring mbTK activity were added to the cuvette, and the reaction was initiated by adding a mixture of substrates. The Michaelis constant was calculated using the dependence of the reaction rate on the cofactor concentration plotted in Lineweaver– Burk coordinates.

Substrate binding constants were determined using a standard method by varying the substrate concentration within the ranges of 0–100 and 0–215 µM for X5P and R5P, respectively. The concentration of the second substrate was constant: 320 μM. The Michaelis constants were calculated by plotting the dependence of the reaction rate on substrate concentration in Lineweaver–Burk coordinates.

To compare the active centers of TKs from different organisms, we used the crystal structures of mbTK [6], yeast TK (yTK) [10], and human TK (hTK) [11]. The TK sequences were aligned using the Matt 1.0 software [12]. The crystal structures were visualized using the VMD 1.9.2 software [13].

## RESULTS AND DISCUSSION

A recombinant protein for studying the biochemical properties of mbTK was obtained by transforming the *E. coli *strain BL21(DE3) with a plasmid carrying the *Rv1449c *gene. A significant portion (about 50%) of the resulting protein was found to be the apoenzyme, which rapidly loses its activity during isolation and purification. Addition of the TDP cofactor during expression made it possible to increase the holoenzyme content to 75%, which led to enhanced specific activity of the resulting recombinant mbTK, and made it possible to isolate the required amount of active enzyme. It should be noted that the active center of this family of enzymes contains a divalent metal ion: hTK contains a magnesium ion, yTK contains a calcium ion (the metal ion can be replaced when obtaining the TK holoenzyme from the apoenzyme) [9, 14, 15, 16]. The only crystal structure of mbTK available to date contains Mg^2+^ [6]. However, the preferred metal ion under physiological conditions has yet to be determined. When optimizing conditions for obtaining recombinant mbTK, we found that the type of the metal ion (Mg^2+^ or Ca^2+^) used for cultivation and expression does not affect the final yield of the active enzyme.

**Fig. 1:**
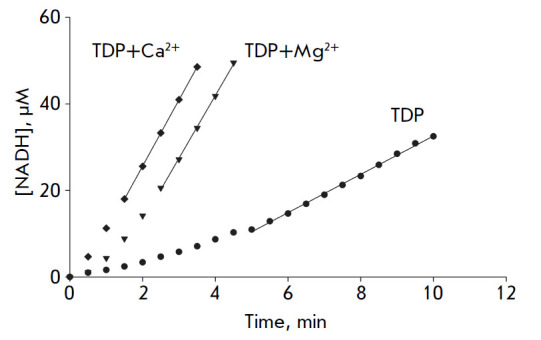
Time dependence of enzymatic activity recovery after the addition of TDP (200 μM) and either Mg^2+^ or Ca^2+^ion (2.5 mM) to the mbTK apoenzyme

The apo form of the enzyme was required to study mbTK affinity to the cofactor. Various methods for cofactor removal have been reported: dialysis, chromatography, and precipitation with ammonium sulfate. In the case of the east enzyme yTK, the cofactor dissociates from the protein during dialysis in a slightly alkaline medium [17], while hTK cofactors can be removed only by precipitation with ammonium sulfate in an acidic medium [9]. We managed to purify mbTK from the cofactor by precipitation with ammonium sulfate in acidic medium (pH 3.5). Apoenzyme activation and proper mbTK function require a simultaneous presence of a metal ion and a TDP molecule in the active center (see *Table 1*). It should be noted that the rates of apoenzyme activation and holoenzyme formation are higher in the presence of Ca^2+^ ions than in the presence of Mg^2+^ ions (*Fig. 1*). In addition, reconstitution of the mbTK holo form in the presence of cofactors is much more efficient at 25°C (compared to 0°C).

**Table 1:** Recovery of enzymatic activity upon activation of the mbTK apoenzyme in the presence and absence of metal ions and TDP

Mg^2+^/Ca^2+^ (2.5 mM)	TDP (200 µM)	Residual activity, %
–	–	5
+	–	5
–	+	30
+	+	100

**Fig. 2:**
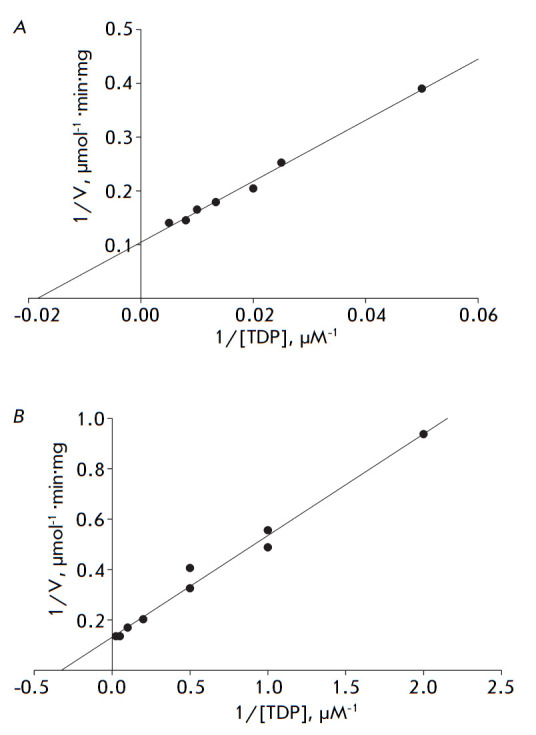
Dependence of the initial rate of the reaction catalyzed by mbTK on the TDP concentration and evaluation of the Michaelis constant in the presence of Mg^2+^ (A) and Ca^2+^ ions (B)

In order to determine the Michaelis constant for TDP, the mbTK apoenzyme was preliminarily incubated in a solution containing a divalent metal ion and the cofactor at different concentrations. The *K*m value was 57 and 3 µM in the presence of Mg^2+^ and Ca^2+^ ions, respectively (*Fig. 2*). It should be noted that mbTK affinity to the cofactor is significantly lower than that of homologous eukaryotic enzymes (*Table 2*). Thus, the *K*m values for TDP were an order and three orders of magnitude lower for yeast yTK and human hTK, respectively. In order to determine what type of interactions in the active site has such a significant effect on the cofactor binding efficiency, we analyzed the structural organization of the cofactor binding sites in the mbTK (3rim), yTK (1ngs), and hTK (3mos) crystal structures.

**Table 2:** K_m_ values for TDP in reactions catalyzed by TKs from different organisms in the presence of Mg^2+^ and Ca^2+^ ions

Enzyme	K_m_ (Mg^2+^), µM	K_m_ (Ca^2+^), µM
hTK	0.074 [9]	not defined
yTK	0.22–4.4 [18]	0.032–0.250 [4, 14]
mbTK	57	3

**Fig. 3:**
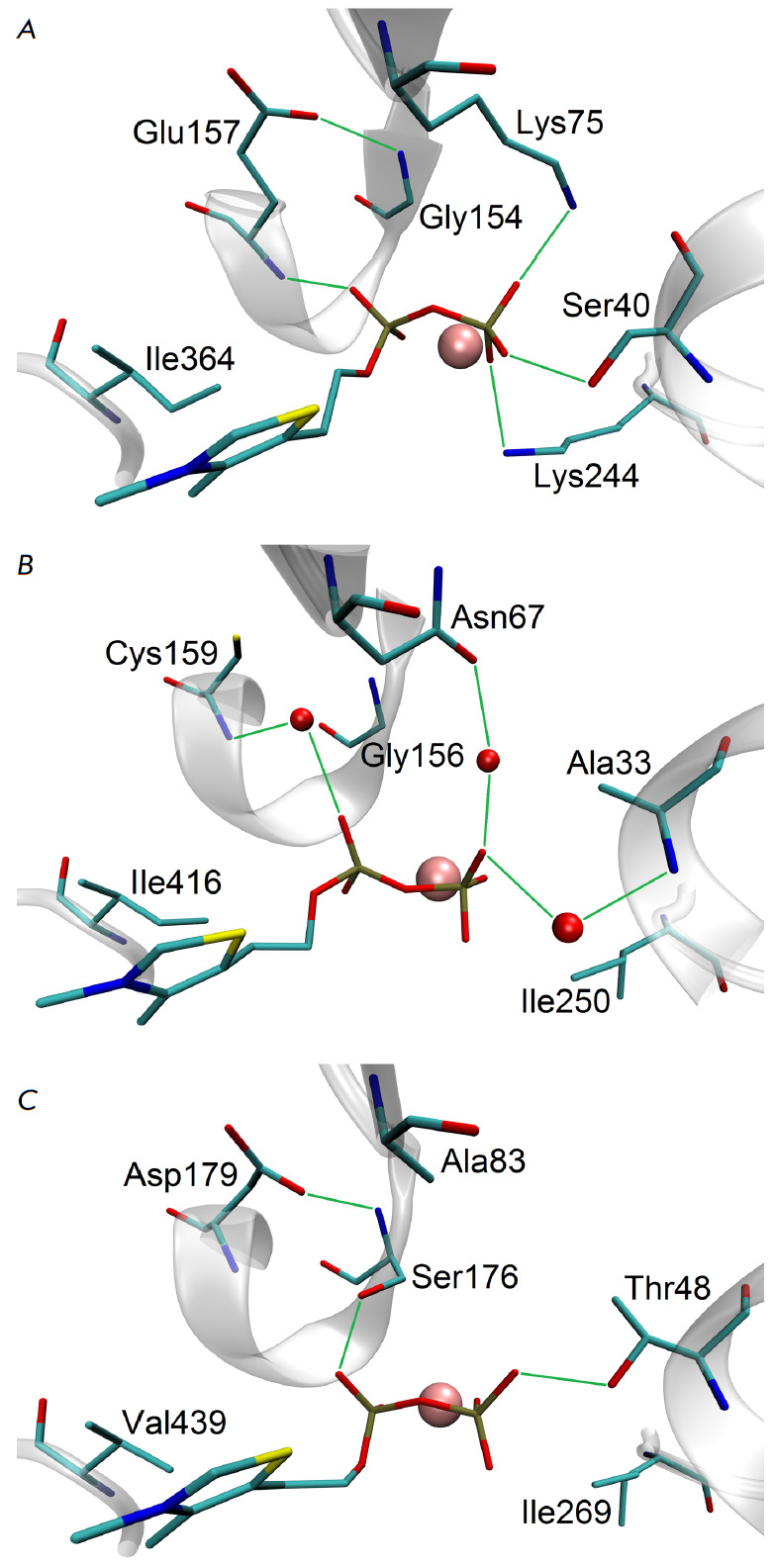
Interactions of the TDP cofactor with variable residues in the active sites of the homologous enzymes hTK (A), yTK (B), and mbTK (C). The pyrimidine fragment of the TDP molecule is not shown. The divalent metal ion is shown in pink; hydrogen bonds are shown in green

In the human enzyme hTK, the Lys75 and Lys244 residues make a significant contribution to the binding energy due to direct electrostatic interaction with the TDP pyrophosphate group. In yTK Lys75 is replaced by Asn67, which interacts with the pyrophosphate group via water molecules, and in mbTK it is replaced by Ala83 that does not interact with TDP \ (*Fig. 3*). The polar residue Lys244 in hTK is replaced by hydrophobic Ile250 in yTK and Ile269 in mbTK. The Ile416 residue in yTK forms a stronger hydrophobic interaction with the thiazole fragment of the TDP molecule compared to Val439 in mbTK (*Fig. 3*). We assume that these substitutions make a key contribution to the decreased affinity to TDP in the series hTK > yTK > mbTK. Meanwhile, a group of variable residues, Ser40/Ala33/Thr48, Gly154/Gly156/Ser176, and Glu157/Cys159/Asp179 (hTK/yTK/mbTK), either directly or indirectly forms two hydrogen bonds with the TDP pyrophosphate group in all three proteins.

The properties of the substrate binding sites in enzymes of differing origin vary less than those of the cofactor binding sites. This conclusion is supported by the *K*m values determined for two substrates, X5P and R5P, in mbTK catalyzed reactions in the presence of magnesium ions. We have studied the dependence of the enzymatic reaction rate on the concentration of one of the substrates in an excess of the second substrate, with the concentration of the second substrate not exceeding the maximum concentration of the variable component by more than 3.5-fold. This limitation was due to possible competition between the substrates for binding to the active site, which was noted for yTK [19]. The obtained *K*m values – 30 μM for X5P and 134 μM for R5P –are comparable with the *K*m values for these substrates in reactions catalyzed by hTK and yTK (*Table 3*), which is consistent with the sequence conservation of the binding site.

**Table 3:** Affinity to the X5P and R5P substrates in reactions catalyzed by TKs from different organisms in the presence of Mg^2+^ ions

Enzyme	K_m_ (X5P), µM	K_m_ (R5P), µM
hTK	11 [9]	63 [9]
yTK	71 [9]	400 [20]
mbTK	30	134

## CONCLUSIONS

We obtained holo, as well as apo, forms of mycobacterial transketolase mbTK using a pET-19b plasmid carrying the *Rv1449c *gene, and isolated and purified the recombinant enzyme. The biochemical characteristics of mycobacterial transketolase mbTK were shown to differ significantly from those of both the homologous human enzyme hTK and the yeast enzyme yTK due to a substitution of lysine residues in the active center by less polar amino acid residues. The affinity of mbTK to the cofactor was found to be almost three orders of magnitude lower than that of hTK. Therefore, it is easier for low-molecular-weight compounds to compete for the TDP binding site in the active center of mycobacterial TK. This feature makes it possible to develop a new class of antibacterial inhibitors that selectively inhibit mbTK activity while exerting no significant effect on hTK.

## Abbreviations

TK: transketolase
hTK: human transketolase
mbTK: mycobacterial transketolase
yTK: yeast transketolase
TDP: thiamine diphosphate
X5P: xylulose 5-phosphate
R5P: ribose 5-phosphate
